# Pulsed laser deposition of ZnO/Mg*_x_*Zn_1-*x*_O superlattices for quantum cascade laser applications

**DOI:** 10.1080/14686996.2026.2701639

**Published:** 2026-07-16

**Authors:** Tatsuya Masuda, Toshihiro Sato, Norihiko Sekine, Iwao Hosako, Gen-Ichi Hatakoshi, Hideomi Koinuma, Ryota Takahashi

**Affiliations:** a Research and Development Department, Smart Combinatorial Technology, Tokyo, Japan; b Design Department, Vacuum Products, Tokyo, Japan; c Beyond 5G Research and Development Promotion Unit Terahertz Technology Research Center, National Institute of Information and Communications Technology, Tokyo, Japan; d College of Engineering, Department of Electrical and Electronic Engineering, Nihon University, Fukushima, Japan

**Keywords:** Pulsed laser deposition, quantum cascade laser, ZnO/Mg*_x_*Zn_1-_*_x_*O superlattices

## Abstract

We developed a precise molecular layer deposition technique using pulsed laser deposition (PLD) to realize a quantum cascade laser (QCL) structure using wide-bandgap semiconductors ZnO and Mg_*x*_Zn_1-*x*_O. Compared to GaAs and InP, ZnO has a larger optical bandgap (3.3 eV) and higher longitudinal optical phonon energy (72 meV). This combination suppresses leakage current and promotes fast nonradiative relaxation, making ZnO a promising material for high-temperature QCL devices operating near room temperature. Nanometer-order thickness control is essential for realizing a QCL device structure. However, PLD techniques suffer from fluctuations in the deposition rate caused by viewport contamination. In this study, a quartz crystal microbalance is used to continuously measure the deposition mass during the PLD process. The combination of a cooling and shielding design with an infrared heating system is used to suppress the thermal drift and plasma damage. Consequently, precise molecular layer control is achieved in the periodic structure of the ZnO/Mg_*x*_Zn_1-*x*_O superlattice. The prepared ZnO QCL structure is characterized by scanning transmission electron microscopy and X-ray diffraction, which confirms that the designed superstructure is reproduced with high precision. This study establishes a PLD process to realize ZnO-based QCL and paves the way for developing new QCL platforms using wide-bandgap materials.

## IMPACT STATEMENT

For quantum cascade laser applications of ZnO/Mg_*x*_Zn_1-*x*_O superlattices, a pulsed laser deposition process was developed that allows precise control of molecular layer thickness using a quartz crystal microbalance.

## GRAPHICAL ABSTRACT

**Figure:**
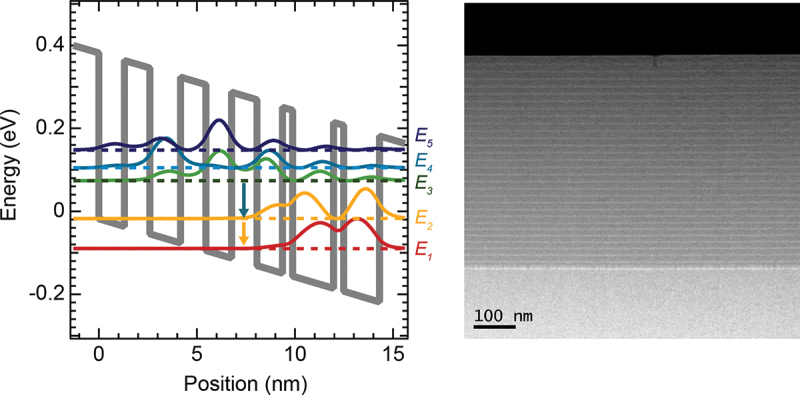

## 1. Introduction

Quantum cascade devices employ a periodic semiconductor quantum well structure to generate optical gain and detect intersubband transitions in quantum wells instead of interband transitions [1]. Quantum cascade lasers (QCL) and quantum cascade detectors have been developed using semiconductor materials such as GaAs [2–5], InP [5], and GaN [6,7], and they operate in the near-infrared range to the relatively long terahertz (THz) wavelength range. QCL emissions in the THz range can penetrate materials that are typically opaque to visible wavelengths, making them suitable for various applications, including various types of detection and medical treatments. This THz light-emitting technology has potential applications beyond 5 G.

A QCL is composed of multiple quantum wells with high-bandgap barrier layers sandwiching low-bandgap well layers [1]. Quantum wells (QWs) are formed by layering materials with different band gaps. The thickness of each layer is on the order of nanometers. Electrons confined within the well create quantized subband energy levels. Quantum cascade devices use quantum wells as a periodic structure inducing intersubband transitions within the well instead of interband transitions, as in conventional semiconductors. The intersubband transition plays an important role in the optical gain.

A schematic diagram of the energy bands in the conduction band of a typical three-level QCL is shown Figure S1. The device structure consists of alternating electron injection sections and active sections; one pair of these sections constitutes a single period. Under an operational bias, the voltage is designed to flatten the minibands within the superlattice of the injection section. This section acts as a vital bridge between adjacent periods: it facilitates the transport of electrons from the depopulated lower level (*E*_1_) of one active section into the upper lasing level (*E*_3_) of the subsequent active section via resonant tunneling. Within each active section, the laser transition occurs between *E*_3_ and *E*_2_ levels. Unlike conventional semiconductor lasers that rely on interband transitions, QCL utilize inter-subband transitions where non-radiative relaxation due to high-speed longitudinal optical (LO) phonon emission is prominent. To maintain a robust population inversion between *E*_3_ and *E*_2_ levels, the energy separation between *E*_2_ and *E*_1_ levels is precisely designed to resonance with the LO phonon energy, ensuring the rapid depopulation of *E*_2_ into *E*_1._ The electrons then enter the next injector, repeating the process across the entire cascaded structure. Repeating these active and injector sections increases the optical gain, enabling QCL operation. Consequently, the total optical gain of QCL devices strongly depends on the superlattice material composition, well width, barrier thickness, and bias. Designing each parameter appropriately is necessary to maximize the optical gain. The lasing wavelength is determined by a quantum-well design instead of the optical bandgap, and therefore, it is possible to design a structure that matches the desired wavelength, thereby enabling flexible control from the mid-infrared to the THz region.

GaAs- and GaN-based superlattices [2–7] were used as QCL devices and emitted light in the near-infrared to the THz wavelength range. In this study, we focused on ZnO-based QCL devices and developed a pulsed laser deposition (PLD) process that enables the atomic-level control of film thickness. Compared to common QCL materials such as GaAs (1.42 eV) and InP (1.35 eV) [1], ZnO has a considerably larger bandgap (3.3 eV) [8–11] comparable to that of GaN (3.4 eV) [6,7]. This large bandgap suppresses the leakage current generated by the thermally excited carriers and enables high-temperature operation. Consequently, QCL devices are expected to operate at high temperatures, near room temperature, or even without cooling. In addition, applying ZnO with a wide bandgap to the QCL increases the insulating properties of the QCL devices, which leads to the formation of a barrier with a low leakage current, which not only strengthens electron confinement but also increases the degree of freedom in designing the quantum level [12,13].

The precise tuning of the bandgap of ZnO films is important for realizing ZnO QCL devices [14–21]. The Mg and Cd ions are well-known dopants suitable for controlling the bandgap of ZnO films. Doping ZnO with Mg^2+^ ions widens the bandgap. Doping with Cd^2+^ ions narrows the bandgap [8–11]. In the Mg_*x*_Zn_1-*x*_O systems, the bandgap increases to 4.5 eV when the Mg content is *x* = 0.47 [22]. Therefore, Mg_*x*_Zn_1-*x*_O/ZnO superlattices form quantum-well structures, which enhance the luminescence efficiency of the photoinduced lasers [19–21]. Similar structures can be applied to QCL devices, potentially providing significant advantages that are unavailable or strictly limited in conventional GaAs- or InP-based systems. A key merit of the Mg_*x*_Zn_1-*x*_O/ZnO heterostructure is the substantially large conduction band offset. This enables the formation of deep quantum wells that thoroughly suppress electron leakage from the upper laser level to higher subbands or the continuum state. Consequently, this advantage allows for efficient operation at shorter wavelengths in the mid-infrared range, as well as stable performance at higher operating temperatures-capabilities that are challenging to achieve with traditional InP- or GaAs-based systems due to carrier leakage and lattice-matching constraints.

Another advantage of applying ZnO films to QCL devices is their high LO phonon energy of 72 meV [17], which is higher than that of GaAs (36 meV) and InP (43 meV) commonly used in QCL devices. Ensuring fast nonradiative relaxation from the upper to the lower levels is important for designing the QCL device. Applying ZnO facilitates accelerated nonradiative relaxation when the intersubband spacing is approximately designed to match its high LO phonon energy, making it easier to maintain population inversion. In addition, even if there is a large energy difference between the active and injector sections, phonons can efficiently dissipate energy, potentially facilitating the design of short-wavelength QCL devices [14].

We note that GaN possesses a large LO phonon energy of 92 meV [6,7], larger than that of ZnO crystals. Consequently, both materials have attracted significant attention for the development of THz QCL devices capable of room temperature operation. A key advantage of employing ZnO over GaN for QCL applications lies in the potentially higher optical gain resulting from the interplay of spontaneous and piezoelectric polarizations. Park et al. investigated the optical gain of quantum well structures using ZnO/Mg_*x*_Zn_1-*x*_O and GaN/Al_*x*_Ga_1-*x*_N systems, reporting that the former exhibits a tendency for spontaneous and piezoelectric polarizations to cancel each other out [23]. This leads to a higher optical gain in the ZnO-based system. Similar phenomena are expected for ZnO QCL. The suppression of the quantum-confined Stark effect leads to the stronger optical transitions with higher overlap of electron wavefunctions, as well as the design flexibility for easier alignment of subband levels, which is critical for complex cascading structures like QCL. Therefore, the reduced internal electric field represents a significant advantage for the realization of high-performance ZnO-based QCL devices on polar substrates.

While molecular beam epitaxy (MBE) has traditionally been the dominant process for ZnO QCL research, this study focuses on the PLD process for device fabrication. During PLD, a significant challenge arises as the viewport, through which the ablation laser introduced, gradually becomes contaminated by the material plume. This fouling alters the deposition rate over time, posing a critical hurdle for the reproducible fabrication of superstructures with nanometer-scale precision. Despite this, numerous oxide-based superlattices have been successfully synthesized via PLD process [24,25]. Excluding ‘natural superlattices’ such as high-temperature superconductors, the fabrication of artificial superlattice through a layer-by-layer growth necessitates precise lattice matching between the film and substrate materials.

When attempting to grow relatively thick superlattices (up to ~500 nm), as required for QCL structures, an inadequate lattice match causes the film to rapidly exceeds the critical thickness for epitaxial strain [26]. This inevitably leads to structural relaxation, resulting in a high density of threading dislocations and other defects. Such structural degradation not only hinders the formation of the target superstructure but also risks obscuring the intrinsic physical properties unique to the superlattice. Furthermore, the limited commercial availability of single crystal oxide substrates often restricts research to specific systems, such as perovskites on SrTiO_3_ [24,25] or ZnO-based superlattices [19–21]. Consequently, achieving precise layer-by-layer growth for a 500-nm-thick superstructure using PLD remains a formidable technical challenge. The extended growth periods necessary for such thickness further exacerbate viewport fouling, which significantly degrades stacking accuracy. Therefore, developing advanced techniques to enhance both film thickness reproducibility and stacking accuracy is essential for advancing superlattice research.

In this study, we designed ZnO and Mg_*x*_Zn_1-*x*_O superlattices to fabricate a QCL structure emitting near-infrared light. Then, we developed a PLD process to precisely fabricate the designed superlattice structure composed of ZnO and Mg_*x*_Zn_1-*x*_O films. The precise control of the film thickness was essential for fabricating ZnO QCL structures. Therefore, we incorporated an in situ film thickness measurement technique using a quartz crystal microbalance (QCM), which was widely used for organic thin films, into PLD to control the deposition thickness in real time and improve the stacking accuracy of the superstructure. Consequently, we succeeded in precisely controlling the nanometer-order film thickness required for fabricating the QCL structure and preparing a complex ZnO QCL structure designed to emit mid-infrared light.

## 2. Experimental section

Prior to film deposition, the ZnO substrate (Z-Crystal LLC, Japan) was ultrasonically cleaned in acetone for 5 min. The ZnO substrate was then annealed in an electric furnace at 1150°C for 3.5 h to obtain an atomically flat surface. During annealing, a ZnO single-crystal substrate was placed on a ZnO ceramic target (Kojundo Chemical Lab. Ltd., Japan, 99.99%) on an alumina crucible to prevent the evaporation of Zn atoms caused by high vapor pressure [27].

After surface treatment, ZnO thin films were deposited via PLD (Vacuum Products: Double Laser PVD, Japan) on the oxygen face of ZnO(000$\overset{ˉ}{1}$) heated by a 1060 nm infrared laser [28,29]. The ZnO(000$\overset{ˉ}{1}$) surface was selected because it has been shown to yield a flatter surface in previous studies [27], making it highly suitable for creating abrupt interface structure. Moreover, the spontaneous and piezoelectric polarizations in ZnO/Mg_*x*_Zn_1-*x*_O on the polar tend to compensate for one another [23]. This makes the ZnO(000$\overset{ˉ}{1}$) surface not only morphologically superior but also electrostatically suitable for QCL operation.

A 266 nm laser pulse equivalent to the fourth harmonic of a neodymium-doped yttrium aluminum garnet (Nd:YAG; LOTIS LS-2145N/4, Belarus) [30,31] was focused on the ceramic targets of ZnO (Kojundo Chemical Lab. Ltd., Japan, 99.99%) and Mg_0.1_Zn_0.9_O (Toshima Manufacturing Co., Ltd., Japan, 99.9%) for laser ablation. Due to the preferential re-evaporation of volatile Zn during the PLD synthesis, the Mg content in the resulting films typically becomes twofold higher than that of the target material [9]. To compensate for this effect, the Mg_0.1_Zn_0.9_O target was used to obtain the intended Mg_0.2_Zn_0.8_O layers.

The laser frequency was 10 Hz, and the fluence was 1.5 J/cm^2^ (laser energy: 7 mJ, spot size: 0.467 mm^2^). During film deposition, the substrate temperature was maintained between 400 and 700°C using a proportional integral derivative control of the infrared laser power. The oxygen partial pressure was fixed at 1 × 10^−5^ Torr. A quartz plate was placed between the target and the viewport to prevent contamination of the viewport during film deposition. A stainless-steel cover was placed over the entire area except for the laser entrance. The quartz plate was rotated during film deposition to minimize contamination caused by plume generation during PLD deposition and laser energy loss.

Monitoring the in situ film thickness is crucial for the PLD synthesis of ZnO QCL thin films. The PLD machine employs a QCM (INFICON: SQM-160, Switzerland), which can measure the thicknesses of organic [32,33] and halide [34,35] films, for growing the oxide thin films. Figure S2 (a) schematically illustrates the spatial arrangement of the QCM sensor, shield, target, and substrate. The QCM measures the mass deposited on the sensor using the principle of change in resonant frequency and estimates the deposited film thickness using the assumed density value. When a QCM is used for the PLD synthesis of oxide thin films, the PLD plasma rapidly damages the QCM sensor. In addition, heat from the substrate heater can heat the QCM sensor, potentially resulting in thermal drift in the measurement values. Therefore, in the PLD system, cooling water is used to maintain a constant QCM temperature; the temperature of the measurement system is kept constant during thin-film deposition. Furthermore, a shield is installed in front of the QCM sensor, as shown in the photograph of Figure S2 (b), to minimize damage from the PLD plasma because the deposition of oxide films requires a higher deposition temperature than that during the synthesis of organic and halide films. The substrate is heated locally using infrared laser light, which insulates the QCM sensor and minimizes the thermal drift of the measured mass. Before the preparation of ZnO QCL film, the QCM sensor was calibrated by the thickness measurement of the ZnO and Mg_0.2_Zn_0.8_O films deposited for 1 hour at the same condition with that for ZnO QCL films. The specific tooling factor for each material was calibrated under the used experimental geometry.

The orientation and lattice constant of the film crystal were characterized by X-ray diffraction (XRD: Rigaku Smartlab, Japan, 45 kV, 200 mA) with Cu Kα radiation. Reciprocal space mapping measurement was performed using an XRD system (Empyrean, Malvern Panalytical, The Netherlands, 45 kV, 40 mA). The surface morphology and thickness of the ZnO QCL films were measured through atomic force microscopy (AFM; Bruker, Dimension Icon, Nanoscope V, USA) and a stylus profiler (DektakXT-A, USA), respectively. The cross-section of the film sample was prepared via mechanical cleavage. The cross-sectional interface structure was imaged through high-angle annular dark-field scanning transmission electron microscopy (HAADF-STEM; JEM-ARM200F, JEOL, Ltd., Japan) at 200 kV. Cross-sectional STEM foils were prepared using a focused ion beam and observed from the <11$\overset{ˉ}{2}$0 > direction of the ZnO crystal.

Raman spectroscopy (Horiba, LabRam HR Evolution, Japan) was used to observe the strain induced by the ZnO QCL superstructure. Considering the influence of the superposition of the superstructure and substrate components on Raman observations, the ZnO QCL film was deposited on Al_2_O_3_(0001) substrate instead of ZnO substrate. QCL structures with ZnO and Mg_0.2_Zn_0.8_O were fabricated using PLD with ZnO and Mg_0.1_Zn_0.9_O targets. A 532 nm laser diode was focused on the film sample to a spot size of 1 μm, and the Raman spectra were detected by a liquid nitrogen-cooled charge-coupled device.

## 3. Results and discussion

**Figure 1.**
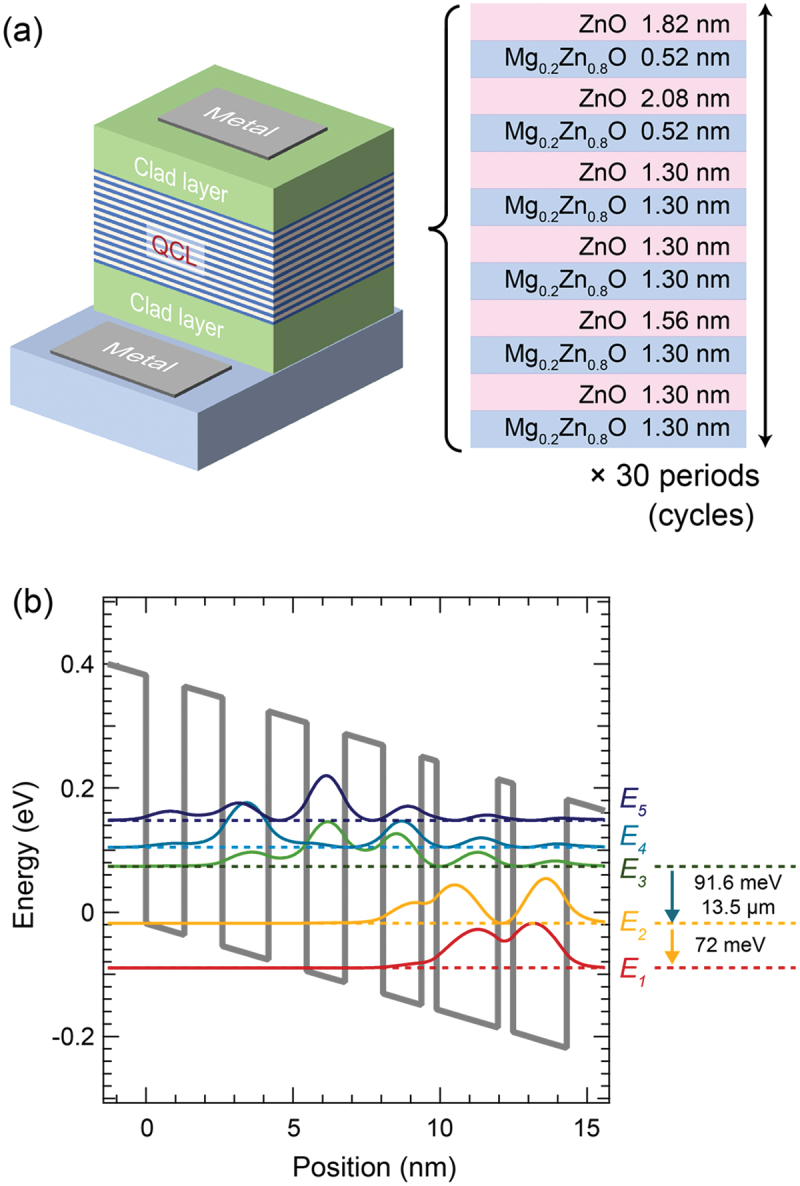
(a) Sample structure of the ZnO QCL device. (b) Conduction band profile and associated square wave function for the ZnO QCL structure biased at 140 kV/cm.

Figure 1(a) shows the structure of the ZnO QCL device. The lower cladding, active, and upper cladding layers were stacked on the ZnO(000$\overset{ˉ}{1}$) O-face substrate. In a QCL, an electric field is applied between the upper and lower electrodes, and a current is injected. The injected electrons undergo inter-subband transitions in the active layer, resulting in a mid-infrared light gain [14–18]. The laser light wavelength can be tuned by appropriately designing the quantum wells in the active layer. The active layer is composed of a quantum-well structure of ZnO and Mg_0.2_Zn_0.8_O. We attempted to fabricate the ZnO QCL structure illustrated on the right side of Figure 1(a). The lattice constant in the *c*-axis direction of ZnO is 0.52 nm [36], the minimum thickness of each layer is set to one unit cell, and the thickness of each layer is set to a multiple of half unit cells.

Figure 1(b) shows the calculated conduction band profile and the squared moduli of the wavefunctions for the designed ZnO QCL structure. The calculation was performed by solving the Schrödinger equations under a biased condition of 140 kV/cm, using typical physical constants for ZnO. In this analysis, the electric field is assumed to be applied uniformly across the 30 period active region to evaluate the fundamental electronic states and transition energies. The photons are emitted upon transition from the upper active levels (*E*_3_–*E*_5_) to the *E*_2_ level. To ensure efficient population inversion, the energy difference between *E*_1_ and *E*_2_ levels was designed to be 72 meV, which resonates with LO phonon energy of ZnO. This design facilitates the rapid population of the *E*_2_ levels. As the primary focus of this study is the design and electronic properties of the QCL active region, the calculated field of 140 kV/cm represents the effective field strength within the superlattice required to achieve a gain at 13.5 μm, which corresponds to the energy difference between *E*_3_ and *E*_2_.

**Figure 2.**
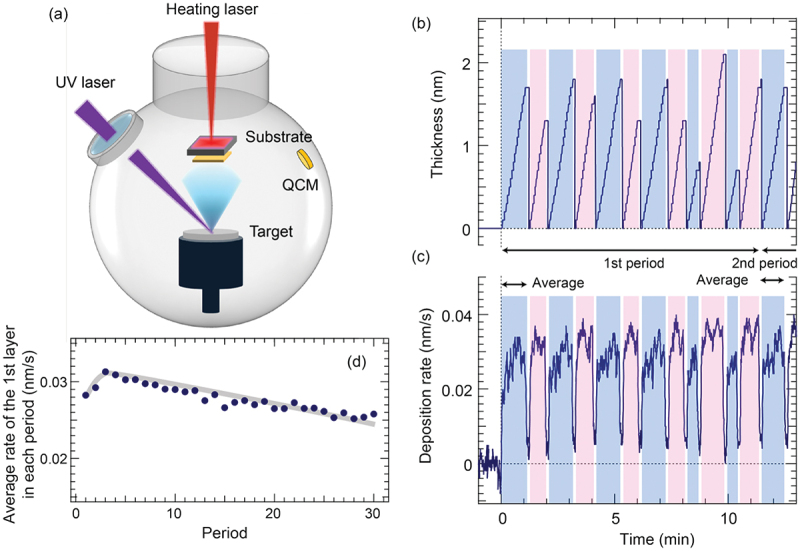
(a) Schematic of the PLD equipment for fabricating the ZnO QCL structures. (b, c) in situ measurements of the film thickness (b) and deposition rate (c), respectively, for each ZnO or Mg_0.2_Zn_0.8_O layer. The pink and blue hatched regions represent the ZnO and Mg_0.2_Zn_0.8_O layers, respectively. (d) The average deposition rate of the first Mg_0.2_Zn_0.8_O layer in each period.

The PLD apparatus shown in Figure 2(a) was applied to the fabrication of the ZnO-QCL structure to precisely fabricate the ZnO QCL structure. The most important factor when fabricating a ZnO QCL structure using PLD is the precise control of the film thickness. For PLD, the ablated plasma contaminates the viewport through which the UV laser beam is incident, and it depends on the ablated material. This contamination can be severe depending on the material, significantly reducing the energy of the UV laser [37]. Consequently, the deposition rate of the film changed significantly between the start and end of film deposition. In the case of the ZnO QCL, the well width differed near the interface with the substrate and near the film surface. This made it difficult to create the designed superlattice structure [27].

As schematically illustrated in Figure 2(a), a QCM sensor was installed in the PLD system to precisely control the thicknesses of the ZnO and Mg_0.2_Zn_0.8_O films. The sensor was used for the in situ measurement of the deposited film thickness. The computer detected the film thickness and automatically stopped laser ablation when the desired thickness was reached. Figure 2(b,c) show the deposition time evolution of the film thickness (b) and deposition rate (c) of each layer immediately after the start of the synthesis of the designed ZnO QCL. The deposition automatically stopped after a 1.3 nm-thick Mg_0.2_Zn_0.8_O thin film layer was deposited. Subsequently, the ablation target was changed to ZnO, and the 1.3-nm-thick ZnO film was deposited as the second layer. Figure 1(a) shows that one ZnO QCL cycle comprises 12 layers. This sequence was repeated 30 times. The deposition rates are shown in Figure S3. The deposition of the ZnO QCL film shown in Figure 1 took ~6 h.

Viewport contamination is a serious problem during the deposition of ZnO QCL. The impact on PLD deposition is investigated using the deposition rate immediately after the deposition to the end of the deposition, as shown in Figure S3. A gradual decrease in the deposition rate was clearly observed. Figure 2(d) shows the calculated average deposition rate for a 30 period ZnO QCL. The average growth rate was calculated and plotted for the first Mg_0.2_Zn_0.8_O film layer of each period, as shown in Figure 2(c). During the first three periods, the deposition rate increased from 0.028 to 0.032 nm/s. Before the deposition began, the Nd:YAG laser used for ablation did not emit laser pulses, resulting in a slight decrease in the laser temperature compared to that during deposition. Therefore, the laser output energy decreased immediately after the deposition. After the deposition began, the temperature of the Nd:YAG laser increased gradually as the laser irradiated the 10 Hz pulses. The effect of the laser system temperature is shown in Figure 2(c). After the target was replaced, the deposition rate of each layer increased slightly after film deposition started.

However, the temperature effect of the PLD system on the deposition rate weakened after the third cycle, while the effect of contamination on the viewport increased. Therefore, the laser intensity density in the chamber decreased gradually as ZnO QCL deposition progressed. The deposition rate for the third layer was 0.032 nm/s, whereas that for the 30th period was 0.025 nm/s, which was a 22% decrease. In fact, before deposition, the laser energy incident on the target was measured at 7 mJ. However, after the growth of the ZnO QCL film, it was confirmed that the energy decreased to 6 ~ 6.5 mJ due to contamination of the viewport. Despite this attenuation, the laser energy remained above the threshold required for target ablation, proving sufficient for a continuous 6 hour deposition process. The attenuation suggests that a deposition system that can precisely control the thickness of a superlattice structure, such as a ZnO QCL, using PLD while simultaneously monitoring the thickness of each layer is required.

**Figure 3.**
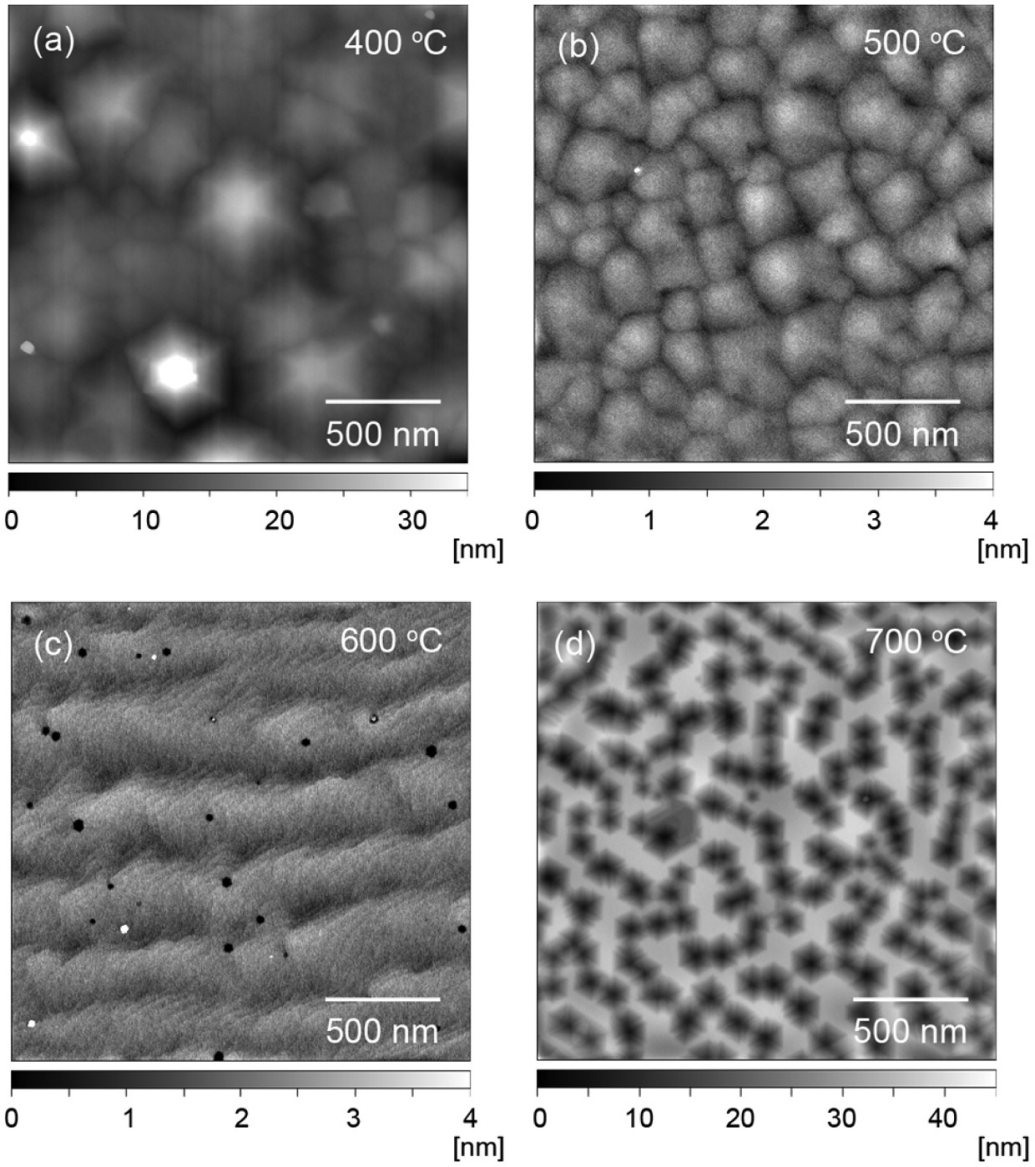
AFM images of the ZnO QCL films grown on the ZnO(000$\overset{ˉ}{1}$) substrate at (a) 400, (b) 500, (c) 600, and (d) 700°C. The surface roughness was calculated at (a) 4.36, (b) 0.47, (c) 0.49, and (d) 7.39 nm.

Figure 3 shows the AFM results of the surface morphology used to estimate the optimal deposition temperature of the ZnO QCL films. The film deposition was performed at 400, 500, 600, and 700°C. The total period was fixed at 30 through in situ QCM measurements. The surface of the ZnO QCL film grown 400°C (Figure 3(a)) exhibits hexagonal pyramidal island growth caused by the hexagonal crystal structure of ZnO, with the gaps between the island-like growth forming grain boundaries. The surface was nonuniform and had a roughness, which means the root mean square height, of 4.36 nm. Surface diffusion was enhanced when the deposition temperature increased from 400°C to 500°C and 600°C. This resulted in a smoother surface. At 500°C, the surface roughness was 0.47 nm, which is a tenth of the value observed for 400°C. Although the sample deposited at 600°C exhibited some small holes, it had the largest flat surface and a step structure derived from the ZnO single-crystal, with a roughness of 0.49 nm. These results confirm that 600°C is the optimal temperature for depositing a layered structure such as a ZnO QCL film. In addition, hexagonal pyramidal pits appeared on the flat surface when the substrate temperature was increased to 700°C, resulting in a high surface roughness of 7.39 nm, making it the roughest surface among the three films. Similar hexagonal pyramidal pits appeared for uniform ZnO films homoepitaxially grown on ZnO(000$\overset{ˉ}{1}$) substrate at 700°C [27]. Although the O-terminated ZnO(000$\overset{ˉ}{1}$) surface is expected to be stabilized by reconstructions under equilibrium conditions [38], the growth is performed at 700°C under nonequilibrium conditions. In this regime, Zn desorption becomes significant, leading to the formation of Zn-deficient regions. Due to kinetic limitations, these regions do not reorganize into long-range ordered reconstructions but instead evolve into pit structures. Furthermore, although hydrogen gas was not intentionally introduced during PLD growth, trace amounts of hydrogen species may exist in the growth environment, originating from residual water vapor in the PLD chamber (under 10^−5^ Torr of oxygen) or the out-diffusion of unintentional hydrogen impurities from the ZnO substrate at the high temperature of 400–700°C. The hydrogen-assisted stabilization is suppressed at high temperature of 700°C, further destabilizing the surface. The hexagonal shape of the pits reflects the intrinsic symmetry of the ZnO lattice and anisotropic step energies. Therefore, the observed pits are attributed to kinetic effects rather than equilibrium surface structures.

**Figure 4.**
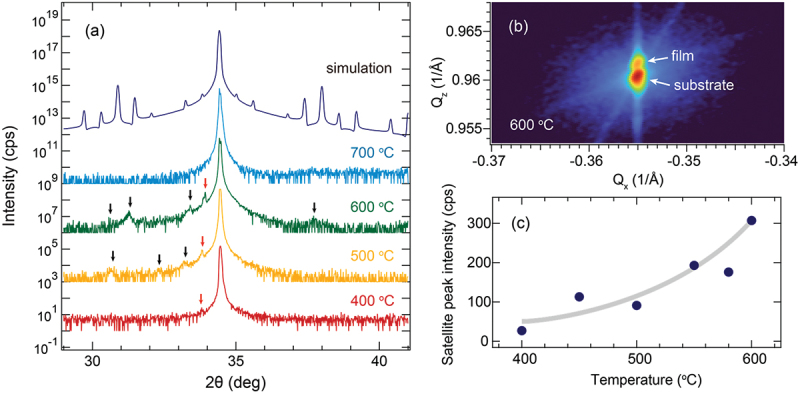
(a) XRD patterns of ZnO QCL films grown on the ZnO substrate at 400 (red), 500 (orange), 600 (green), and 700°C (light blue). The dark blue pattern represents the calculated XRD pattern of the ZnO QCL structure illustrated in Figure 1(a). The arrow indicate that the satellite peak originated from the formation of superlattice structures. (b) Reciprocal space mapping around the ZnO -105 peak for the ZnO QCL film grown at 600°C. (c) Growth temperature dependence of the satellite peak intensity marked by the red arrows in (a).

Figure 4(a) shows the XRD patterns around the 002 peak for ZnO QCL films deposited at 400, 500, 600, and 700°C. The simulated XRD pattern of the ZnO QCL structure in Figure 1(a) is shown in the upper part of Figure 4(a). Peak splitting of the ZnO 002 reflection is seen in the films grown at temperatures excluding 400°C. This phenomenon originates from a reduction in the *c*-axis lattice parameter induced by Mg substitution, which causes a slight lattice mismatch between the average superlattice structure and the ZnO substrate. Figure 4(b) shows the reciprocal space mapping around the ZnO -105 reflection for the ZnO QCL film deposited at 600°C. The film peak is clearly resolved from the ZnO substrate peak. The in-plane lattice constant of the ZnO QCL film is identical to that of the ZnO substrate, indicating coherent growth of the ZnO QCL layer. For the ZnO QCL film deposited at 700°C, which had the roughest surface determined by AFM, only the ZnO 002 peak was observed, and it showed no satellite peaks originating from the superlattice structure. This indicates that the ZnO QCL film is not deposited at the optimal temperature, thereby resulting in a disordered superstructure or a uniform Mg concentration. In the XRD patterns of the ZnO QCL films deposited at 400, 500, and 600°C, small satellite peaks originating from the superlattice were observed in addition to the ZnO 002 peak. Figure 4(c) shows the intensity of the satellite peak closest to the ZnO 002 reflection, indicated by the red arrow in Figure 4(a), plotted as a function of the substrate temperature. With an increasing substrate temperature, the satellite peak intensity increased gradually, indicating an increased order in the superstructure.

We note that the satellite peaks originating from the superstructures in ZnO QCL films were observed only at the low angle side. In ideal superlattices, satellite peaks typically appear symmetrically around the main Bragg peak. However, P. Bodeker et al. reported that satellite peaks weaken at high angles in Co/Cu superlattices [39], similar to the results in Figure 4(a). This phenomenon can be attributed to the fact that Co is stretched along the in-plane direction while Cu is compressed, inducing a strong Poisson strain in the thickness direction. Furthermore, interface roughness and broadening may further contribute to the asymmetric satellite peaks. High-angle diffraction peaks are more sensitive to small-scale structural fluctuations; thus, the presence of interfacial grading or roughness acts similarly to a Debye-Waller factor, causing a more pronounced attenuation of high-order and higher-angle reflections. Therefore, one possible explanation for the asymmetric satellite peaks in Figure 4(a) is the inherent effects of the ZnO QCL films, including the strong strain and interface roughness. While a comprehensive quantitative XRD simulation is required to fully clarify the structural model (including effects such as interface roughness, composition grading or period fluctuations), the strain-induced phase shift in the diffracted wave provides a plausible explanation for part of the observed peak asymmetry.

**Figure 5.**
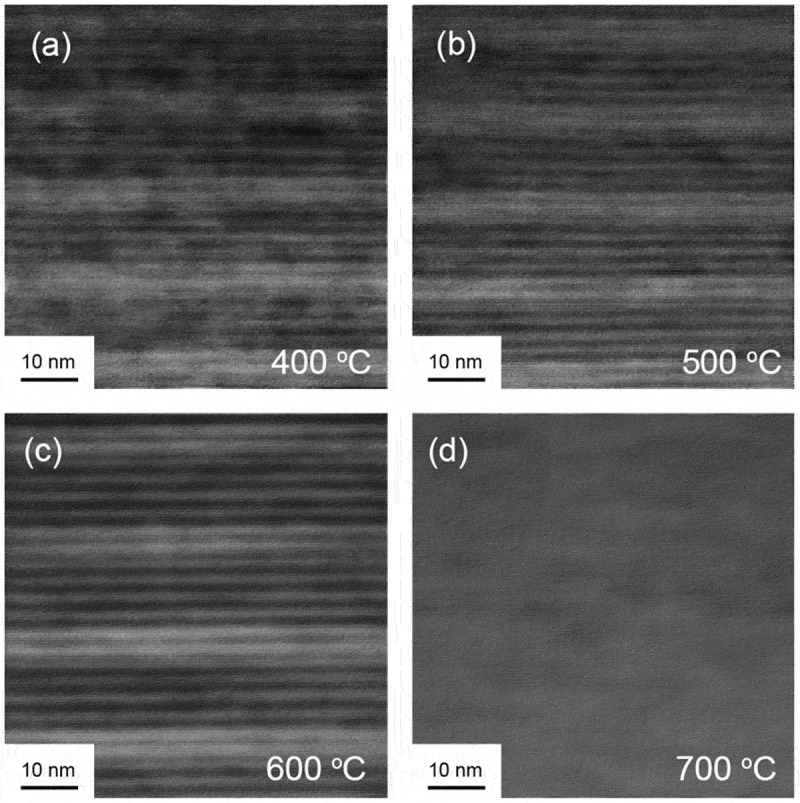
HAADF-STEM images of the ZnO QCL films grown on the ZnO substrate at 400 (a), 500 (b), 600 (c), and 700°C (d).

Furthermore, the positions of the satellite peaks do not necessarily coincide with the simulations. While the presence of these peaks indicates the formation of a superlattice, XRD analysis alone was insufficient to determine whether the target structure shown in Figure 1 was precisely synthesized via the PLD method. Therefore, the periodic structure of the superlattice was investigated through HAADF-STEM. The Z contrast in HAADF-STEM images is highly sensitive to atomic number, making it easy to see the difference in contrast if there is a periodic structure in the range of a few nanometers to a few tens of nanometers, thus making it a meaningful measurement method for confirming the formation of superstructures. Figure 5(a–d) show the HAADF-STEM images of the ZnO QCL films. Elements with higher atomic numbers exhibit stronger scattering, which results in more electrons entering the detector and appearing brighter in the STEM image. In the HAADF-STEM images of the ZnO QCL, the Mg_0.2_Zn_0.8_O layer containing light Mg appeared dark, while the ZnO layer that did not contain Mg appeared whiter. Contrast differences in STEM images resulting from the superlattice became clearly observable with increasing deposition temperature from 400°C to 500°C and 600°C, reflecting improved layer/interface definition of superstructure and consistent with the increase of satellite peak intensity, as shown in Figure 4(c). In contrast, the ZnO QCL film deposited at 400°C, which had the smallest satellite peak characteristic of a superlattice, had a more random superstructure than that of the other two samples grown at 500 and 600°C. In addition, the ZnO QCL film deposited at a higher temperature of 700°C, which activated the diffusion of the Mg dopant at the interface between Mg_0.2_Zn_0.8_O and ZnO, was a uniform film in which Mg was doped into the ZnO film; no difference in contrast was observed for the superstructure. The energy dispersive X-ray spectroscopy (EDS) images of the ZnO QCL film deposited at 700°C confirmed a uniform Mg distribution throughout the ZnO QCL film, as shown in Figure S4. In contrast, the EDS maps in Figure S5 for the film deposited at 600°C clearly demonstrates the periodic Mg compositional modulation within the superlattice structure. This direct comparison provides strong evidence that while the superlattices are successfully maintained at 600°C, significant interlayer diffusion occurs at 700°C, leading to the collapse of the layered structure.

**Figure 6.**
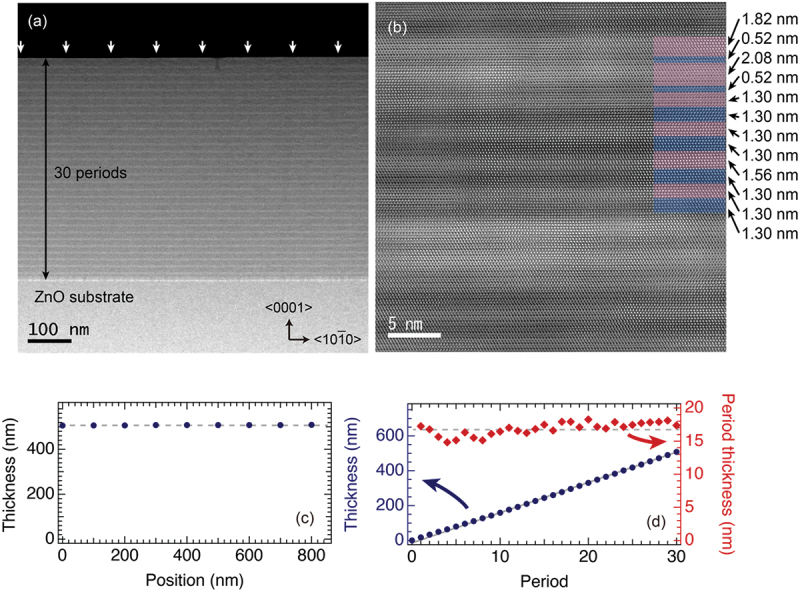
STEM analysis of the ZnO QCL film grown at 600°C. (a) Wide range HAADF-STEM image. (b) Magnified part of the ZnO and Mg_0.2_Zn_0.8_O multilayer structure. The pink and blue hatched regions represent the ZnO and Mg_0.2_Zn_0.8_O layer, respectively. (c) Total film thickness measured at the positions indicated by the white arrows in (a). (d) Total thickness (left) and individual period thickness (right) as a function of period number.

Figure 6(a) shows an HAADF-STEM image measured over a wide area of a ZnO QCL film deposited at 600°C to understand the structure of the ZnO QCL film in more detail. The density of ZnO was higher than that of Mg_0.2_Zn_0.8_O, and therefore, the ZnO layer appeared white. In Figure 6(a), the number of white layers is 30, which clearly demonstrates the periodicity consistent with the period number formed by PLD. Figure 6(b) shows a zoomed-in image of a single period consisting of 12 layers, which clearly demonstrates the structure designed in Figure 1.

The total film thickness is measured from the HAADF-STEM image shown in Figure 6(a) to confirm the precision of PLD film thickness control. Figure 6(c) shows a plot of the total film thickness versus the measurement positions presented by the white arrows in Figure 6(a). The film thickness was uniform over a wide area, with an average thickness of 507.3 ± 1.6 nm. This value is nearly identical to the designed value of 468 nm for the ZnO QCL, as shown in Figure 1. Figure 6(d) plots the thickness from the ZnO substrate surface to each layer calculated from the STEM image versus the period number. The total thickness increases proportionally with the period number. The average thickness per period was calculated from the slope and estimated to be 16.88 ± 0.93 nm. In the magnified STEM image in Figure 6(b), the number of atoms per layer is 65, with an average thickness of 16.88 nm. The reproducibility of ZnO QCL film growth at 500°C is presented in Figure S6. Although the abruptness of the layer interface is slightly reduced compared to growth at 600°C, the controllability of the layer thickness remains excellent, demonstrating consistent reproducibility. Furthermore, STEM measurements confirmed that the physical thickness of the periods in the ZnO QCL film grown at 600°C was indeed greater than that at 500°C. In Figure 4(a), the angular distance between the ZnO 002 main peak and the first observable satellite peak on the low angle suggests that the ZnO QCL film grown at 600°C has a longer periodicity than that at 500°C, which is consistent with the STEM analysis data.

These values demonstrate the highly accurate nanometer-order stacking compared to the design values (15.6 nm, 60 atoms). The film was synthesized using time-controlled deposition at a pre-measured deposition rate when fabricating the ZnO and Mg_0.2_Zn_0.8_O superlattices using conventional PLD [27]. The energy output of the pulsed laser decreased over time toward the surface, significantly decreasing the deposition rate and resulting in a thinner layer. As shown in Figure 2(d), a 22% decrease in deposition rate was clearly observed when comparing the deposition rate immediately after the start of deposition with that at the end of deposition. However, the deposition time can be automatically adjusted to the set film thickness even if the energy output of the laser used for ablation decreases using a QCM film thickness meter. This leads to a significant improvement in repeatability control when fabricating atomic-layer-level stacked structures such as QCL films.

The HAADF-STEM analysis revealed a clear interface between ZnO and Mg_0.2_Zn_0.8_O and a highly crystalline layered structure. If a superstructure of ZnO and Mg_0.2_Zn_0.8_O is deposited at a thickness of 500 nm, a large amount of stress accumulates inside the ZnO QCL film. Such a large strain was also suggested by the asymmetric satellite peaks in XRD patterns shown in Figure 4(a). Therefore, Raman spectroscopy was performed to investigate the stress states. However, the Raman spectra of the ZnO and Mg_0.2_Zn_0.8_O superlattices on the ZnO substrate could not distinguish between the spectra of the individual components of the substrate and the thin film. To complementarily investigate the strain of each ZnO layer within the ZnO QCL superstructure, a ZnO QCL film was fabricated on an α-Al_2_O_3_(0001) substrate. The primary intention for using the α-Al_2_O_3_(0001) substrate was to investigate the strain distribution within the ZnO/Mg_0.2_Zn_0.8_O superlattice layers as auxiliary reference data, rather than focusing on the epitaxial strain from the substrate itself. Due to the significant lattice mismatch between α-Al_2_O_3_ and ZnO, the epitaxial strain from the substrate is largely relaxed within the initial buffer layers. This allowed us to use Raman spectroscopy to selectively observe the internal strain effect arising from the lattice mismatch between the alternating ZnO and Mg_0.2_Zn_0.8_O layers.

**Figure 7.**
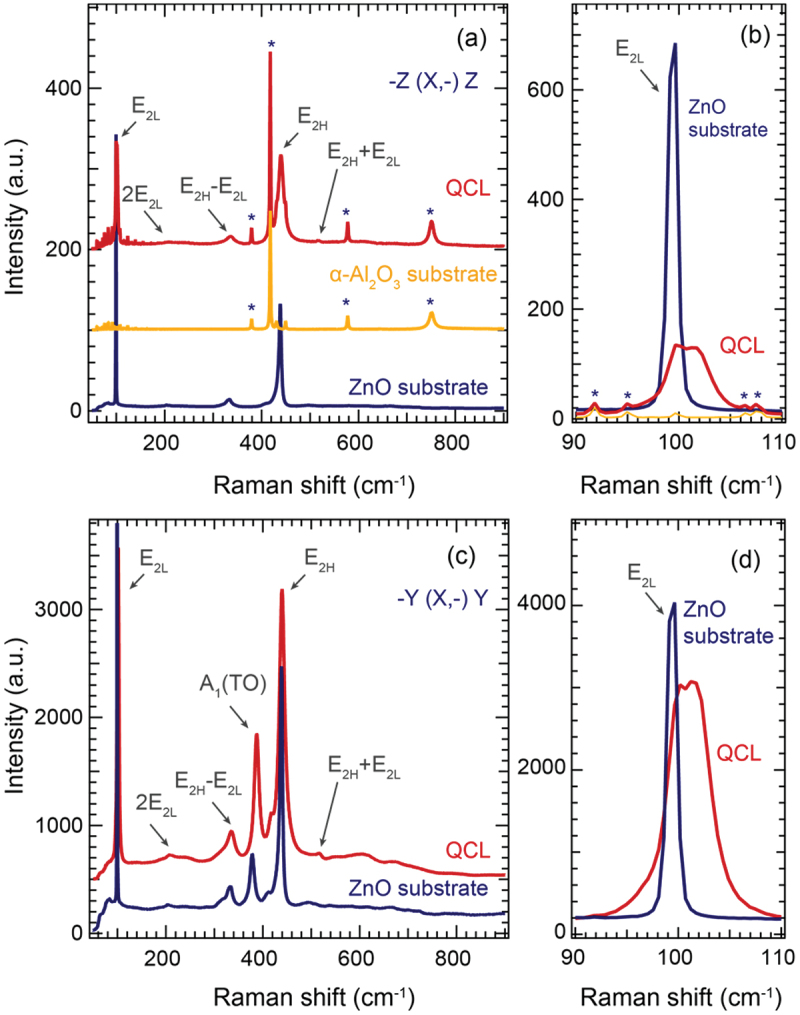
Raman spectra in -Z(X,-)Z (a-c) and -Y(X,-)Y (d-f) configurations for the ZnO QCL film (red) grown on the α-Al_2_O_3_(0001) substrate. (b) and (d) magnified view of the *E*_2 L_ peak near 100 cm^−1^. for comparison, the Raman spectra of α-Al_2_O_3_(0001) (yellow) and ZnO (000 $\overset{ˉ}{1}$) (blue) substrate are also shown. *represents the Raman mode originated from the α-Al_2_O_3_(0001) substrate.

Figure 7 shows the Raman spectra of a ZnO QCL film on an α-Al_2_O_3_(0001) substrate measured in the -Z(X,-)Z direction (a,b) and the -Y(X,-)Y direction (c,d). To distinguish between the components originating from the substrate and film parts, the Raman spectra of the α-Al_2_O_3_(0001) and ZnO(000$\overset{ˉ}{1}$) substrates are shown in Figure 7. In addition to the peaks originating from the α-Al_2_O_3_(0001) substrate, the ZnO QCL film spectra exhibits the *E*_2 L_ (Figure 7(b)) modes around 100 cm^−1^. The *A*_1_(TO) mode Raman peak was forbidden in the -Z(X,-)Z direction and only observed in the -Y(X,-)Y direction. Other modes originating from higher-order Raman lines are also observed.

The *E*_2 L_ mode peaks shown in Figure 7(b,d) exhibit narrower linewidths compared to other phonon modes, allowing the precise characterization of Raman shifts. It has been reported that the substitution of Zn ions with Mg ions in the ZnO lattice increases the *E*_2 L_ mode frequency, leading to a blue shift toward higher wavenumbers [40]. Furthermore, the *E*_2 L_ mode in ZnO crystals is known to shift toward lower wavenumbers under compressive strain and toward higher wavenumbers under tensile strain [41].

In the Raman spectra of the ZnO QCL structure, two distinct peaks were observed; the peaks at lower and higher wavenumbers are assigned to the ZnO and Mg_0.2_Zn_0.8_O layers, respectively. The ZnO related peak exhibits a blue shift relative to the ZnO substrate peak, suggesting the presence of tensile strain within the ZnO layer. Given that the in-plane lattice constants for bulk ZnO and Mg_0.2_Zn_0.8_O are a = 3.250 Å and a = 3.258 Å, respectively [8,9], these results confirm that the ZnO layers experience tensile strain while the Mg_0.2_Zn_0.8_O layers are under compressive strain. These Raman observations are consistent with the strain states predicted from the lattice mismatch, confirming that the *E*_2 L_ mode serves as a reliable indicator of the strain distribution in the QCL film.

The Raman analysis of the ZnO/Mg_*x*_Zn_1-*x*_O superlattice QCL structure complementarily demonstrated the presence of alternating tensile and compressive strains in the ZnO and Mg_*x*_Zn_1-*x*_O layers, respectively. Generally, in lattice-mismatched film structures, strain accumulates monotonically with the number of periods, eventually exceeding the critical thickness and inducing dislocations that degrade the crystallinity of QCL films. However, the large-area cross-sectional STEM image in Figure 5(a) reveals no such dislocations, with high crystallinity maintained throughout the entire ZnO QCL film. This is further supported by the smooth surface structure morphology in the AFM image in Figure 3(c). Crucially, the reciprocal space mapping in Figure 4(b) evidenced the coherent growth without significant strain relaxation, as the in-plane lattice parameters remained locked to those of the ZnO substrate. These results provide a comprehensive explanation for the suppression of strain-relaxation defects: the alternating strain profile acts as a strain-balancing mechanism. By alternating tensile and compressive layers, the net elastic energy of the superlattice is effectively minimized, remaining well below the threshold for dislocation nucleation despite the large total thickness.

The nanometer-precision control of quantum well stacking using the PLD technique developed in this study confirms that it can be a precision synthesis necessary for wide-bandgap materials and high-temperature QCL devices to replace GaAs- and InP-based QCL devices; this is important in oxide QCL research. Moreover, this thin-film process is expected to be useful for the PLD synthesis of superstructures related to various materials, not limited to ZnO films.

## 4. Conclusion

We developed a precision molecular layer deposition technique compatible with oxide materials to form superlattices with nanometer precision. The thermal stabilization and protection design of the QCM measurement system enabled continuous film thickness monitoring under PLD conditions, demonstrating the atomic-scale control of the quantum well and barrier layers. XRD, AFM, and Raman characterization of the resulting Mg_*x*_Zn_1-*x*_O/ZnO superlattice revealed that its crystallinity, periodicity, and strain state matched the designed values. In addition, quantum-level calculations showed that the material system supports effective inter-subband transitions in the mid-infrared region, strongly supporting the idea that ZnO-based wide-bandgap materials are promising candidates for next-generation high-temperature operation QCL devices.

These results pave the way for a new QCL technology based on oxide semiconductors, providing a foundation for achieving high-power, high-temperature, and durable operation, which is difficult to achieve with conventional III-V materials. The film formation and film thickness control techniques established in this research can be applied to oxide quantum devices and are expected to significantly affect future research in photonics and infrared devices.

## Data Availability

The data that support the findings of this study are available from the corresponding authors upon reasonable request.
